# Detecting Genetic Association of Common Human Facial Morphological Variation Using High Density 3D Image Registration

**DOI:** 10.1371/journal.pcbi.1003375

**Published:** 2013-12-05

**Authors:** Shouneng Peng, Jingze Tan, Sile Hu, Hang Zhou, Jing Guo, Li Jin, Kun Tang

**Affiliations:** 1 Human Functional Genetic Variation Group, CAS-MPG Partner Institute for Computational Biology, SIBS, Shanghai, China; 2 State Key Laboratory of Genetic Engineering and Ministry of Education Key Laboratory of Contemporary Anthropology, School of Life Sciences, Fudan University, Shanghai, China; UC Berkeley, United States of America

## Abstract

Human facial morphology is a combination of many complex traits. Little is known about the genetic basis of common facial morphological variation. Existing association studies have largely used simple landmark-distances as surrogates for the complex morphological phenotypes of the face. However, this can result in decreased statistical power and unclear inference of shape changes. In this study, we applied a new image registration approach that automatically identified the salient landmarks and aligned the sample faces using high density pixel points. Based on this high density registration, three different phenotype data schemes were used to test the association between the common facial morphological variation and 10 candidate SNPs, and their performances were compared. The first scheme used traditional landmark-distances; the second relied on the geometric analysis of 15 landmarks and the third used geometric analysis of a dense registration of ∼30,000 3D points. We found that the two geometric approaches were highly consistent in their detection of morphological changes. The geometric method using dense registration further demonstrated superiority in the fine inference of shape changes and 3D face modeling. Several candidate SNPs showed potential associations with different facial features. In particular, one SNP, a known risk factor of non-syndromic cleft lips/palates, rs642961 in the *IRF6* gene, was validated to strongly predict normal lip shape variation in female Han Chinese. This study further demonstrated that dense face registration may substantially improve the detection and characterization of genetic association in common facial variation.

## Author Summary

Heritability of human facial appearance is an intriguing question to the general public and researchers. Although it is known that some facial features are highly heritable, the exact genetic basis is unknown. Previous studies used simple linear measurements such as landmark distances, to evaluate the facial shape variation. Such approaches, although easy to carry out, may lack statistical power and miss complex morphological changes. In this study, we utilized a new 3D face registration method that enables subtle differences to be detected at high resolution 3D images. Based on this, we tried to test and characterize the associations of 10 candidate genetic variants to common facial morphological variations. Different types of phenotype data were extracted and compared in the association tests. Our results show that geometry based data performed better than simple distance based data. Furthermore, high density geometric data outstood the others in capturing small shape changes and modeling the 3D face visualization. Interestingly, a genetic variant from *IRF6* gene, which is also a well-known risk factor of non-syndrome cleft lip, was found to strongly predispose the mouth shape in Han Chinese females.

## Introduction

The human face plays an essential role in everyday life. It hosts the most important sensory organs and acts as the central interface for expression, appearance, communication and mutual identification. Inheritance of facial appearance from parents to their offspring is a constantly intriguing question to the public and scientific community. Indeed, human facial morphology is highly heritable. Twin studies have shown that heritability of facial features is as high as 80% [1], [2]. On the other hand, non-genetic factors also play important roles in shaping the human face, such as age and climate [2]–[6]. High heritability suggests that one's facial characters might be predicted to a certain extent, as long as the genetic determinants are identified and their effects fully understood. Face prediction based on genetic profiling, if feasible, may revolutionize forensics [7] and strongly benefit medical diagnosis [8]. However, the influences of common genetic variants on facial morphogenesis are largely unknown. The current understanding of facial morphogenesis has mainly arisen from developmental biology studies in model organisms. Facial morphogenesis involves a coordinated growth of facial prominences in a precise temporal and spatial sequence, which is tightly regulated by many signaling pathways, including the *BMP*, *SHH*, *FGF*, *GHR* and *Wnt/β-catenin* pathways [9]–[16].

In humans, knowledge of the effects of genetic variation on facial morphology has mainly arisen from studies of congenital craniofacial abnormalities. Non-syndromic cleft lip with or without cleft palate (NSCL/P) is the most common congenital craniofacial defect [3], [16], [17]. Great efforts have been made towards identifying the genetic factors that predispose carriers to NSCL/P, and a large number of candidate risk genes have been proposed [17]–[19]. Among these, the IRF6 gene has shown the most convincing and consistent signals for association across many studies [17], [20]–[24]. Many other craniofacial abnormalities can also result from rare genetic disorders, such as Down syndrome, Rubinstein-Taybi syndrome, Sotos syndrome, Bardet-Biedl syndrome and Noonan syndrome [25]–[29]. Nevertheless, these studies have mainly focused on pathological facial morphological changes.

Relatively few studies have attempted to associate genetic polymorphisms to common facial morphological variations. Several non-synonymous changes in the growth hormone receptor (*GHR*) were suggested to affect mandible shape in Japanese and Chinese populations [30]–[32]. Ermakov *et al*. found that a SNP in *ENPP1*, a gene essential in bone physiology, was significantly associated with upper facial height in Chuvashians [33]. In the *FGFR1* gene, a genetic marker was found to be associated with the cephalic index in multiple populations [34]. Interestingly, a recent study examined several high frequency SNPs associated with differential risks of NSCL/P in a few healthy cohorts, and found that two were associated with normal facial shape variation [6]. This suggests that disease risk alleles may also modulate the phenotypes of unaffected carriers, although within a range of normal variation. Subtle shape alteration patterns induced by disease risk alleles, if properly defined, may help to screen carriers of disease alleles, and therefore facilitate disease prevention. In addition to these candidate gene studies, two genome wide association studies (GWAS) have also recently been carried out in Europeans, to search for genetic loci that influence common facial shape variation, and five loci were found to significantly modulate several nose related features [2], [35].

Anthropometric phenotypes, especially facial features, are highly complex and diverse. Traditional phenotype collection involves the manual measurement of specific distances and angles directly on the specimen or subjects, which is infamously tedious and error prone. In recent years, new imaging technologies, have been developed to allow fast and accurate acquisition of three dimensional facial landscapes without direct physical contact with the subject. Such imaging technologies have greatly facilitated human evolutionary analyses of craniofacial phenotypes [4], [5], [35], [36], as well as genetic association studies of human facial morphological variations [2], [6], [35]. However, the analysis post image acquisition still generally involves manual annotation of landmarks on digital images [4], [5], [35], [36]. More importantly, these inter-landmark distances were the most widely used phenotype measurements in the recent genetic studies of human facial morphology [2], [6], [33]–[35]. Inter-landmark based approaches have several problems. First, when pairwise distances are used as phenotypes, the number of phenotypes increases exponentially with that of landmarks, which often results in over conservative *p* values after multiple-testing correction. Second, the information on shape changes that is conveyed by inter-landmark distances is usually obscure. For example, an extended distance between the nasion and nose tip could signal either more pointed or overall bigger nose. Third, the facial shape cannot be fully reconstructed based on pairwise distances and it is, therefore, hard to perceive the biological meaning of the variation in distances. Thus, methods that directly examine the geometrical configuration of shapes are more desirable for general shape analyses. Such methods involve superimposing sample shapes according to their landmarks, followed by multivariate analyses/tests based on landmark coordinates [37]. More recently, new methods have also emerged to better use high resolution geometrical information. Instead of using only the limited number of traditional landmarks, these methods establish high density correspondence for thousands of mathematical landmarks [8] [38] [39]. Based on such methods, rare genetic diseases could be precisely identified and the syndrome effects could be extracted, predicted and visualized in great detail [40]–[42].

In this study, we first applied the method of high resolution 3D image registration to test the potential genetic associations of the complex normal facial variations, and to infer the detailed effects of genetic variants on face. In brief, we applied high density face registration (HDFR) to capture the comprehensive facial variation information of ∼30,000 3D points (referred to as marker points hereafter) [39]. Based on HDFR, three different schemes of phenotype representation were systematically compared for the detection of genetic associations with 10 candidate SNPs. The first scheme used traditional inter-landmark distances; the second represented the face geometrical shapes based on 15 major landmarks; the third is the high density geometric approach that we first proposed in such kind of studies. It uses the complete geometric data of over 30,000 marker points. The high density geometric data was then further used to examine the detailed phenotype changes associated with candidate SNPs.

## Results

We reviewed the literature for candidate SNPs that may be involved in the morphogenesis of the human face.10 SNPs from 4 genes, *ENPP1*, *GHR*, *FGFR1* and *IRF6* were identified and their functional relevance was listed (Table 1). The ectonucleotide pyrophosphatase/phosphodiesterase 1 (*ENPP1*) gene is a key regulator of bone mineralization. Ermakov et al found that the upstream promoter and 3′ un-translated regions in this gene harbor genetic variants associated with the upper facial height and other phenotypes [33]. Four SNPs, rs7773292, rs6925433, rs6569759, rs7754561 that carry the strongest association signals were added to our candidate list. *GHR* is the growth hormone receptor, which plays essential role in the development. Mutations in this gene induce idiopathic short stature and Laron syndrome, marked by a characteristic facial appearance [31]. Several non-synonymous SNPs, including Pro561Thr (rs6184), I526L (rs6180) and C422F(rs6182) were suggested to contribute to mandibular measures in East Asian populations [15], [31], [32]. In this study, we included rs6180 and rs6184 in our study, as they were validated in Han Chinese [32]. *FGFR1*, the fibroblast growth factor receptor 1 plays an important role in facial morphogenesis, and mutations in this gene lead to syndomes associated with facial abnormality, such as the type 1 Pfeiffer syndrome (MIM 101600) and Kallmann syndrome 2 (KAL2) (MIM 147950) [34]. A tagging SNP of this gene, rs4647905 showed moderate signals of association with cephalic index in multiple ethnic groups [43]. We added another tagging SNP rs3213849 to span the full length of *FGFR1*. The Interferon regulatory factor 6 (*IRF6*) plays a critical role in keratinocyte development. Genetic variants of *IRF6*, especially SNP rs642961, were found consistently associated to NSCL/P throughout many candidate gene and GWAS studies [17], [23], [44], [45] As the genetic risk factors of NSCL/P may also contribute to normal facial variation in healthy carriers [16], we enrolled rs642961 into our study. We further included the SNP rs2236907 of *IRF6*, which seems to have a particularly strong signal in Han Chinese [23], [46].

**Table 1:** The 10 candidate SNPs selected from the literature.

SNPs*	Gene	Allele	Chr	Position	Gene full name	Functional relevance
rs7773292	ENPP1	C/T	6	132141454	Ectonucleotide pyrophosphatase/phosphodiesterase 1	1: key regulator of bone mineralization; 2: 5′UTR and 3′UTR variants associated with upper facial height and other phenotypes [33].
rs6925433	ENPP1	A/G	6	132161059		
rs6569759	ENPP1	G/A	6	132174809		
rs7754561	ENPP1	G/A	6	132254387		
rs6180	GHR	A/C	5	42754996	Growth hormone receptor	1:Essential in growth and development; 2: Mutations induce idiopathic short stature and Laron syndrome [31]; 3: Pro561Thr (rs6184) and I526L (rs6180) variants associated with mandibular height in the normal East Asian populations [27] [29].
rs6184	GHR	C/A	5	42755101		
rs4647905	FGFR1	G/C	8	38391699	Fibroblast growth factor receptor 1	1: Important role in facial morphogenesis; 2: Type 1 Pfeiffer syndrome (MIM 101600) and Kallmann syndrome 2 (KAL2) (MIM 147950) [34]; 3: rs4647905 suggested to be associated with cephalic index in different ethnic groups [43].
rs3213849	FGFR1	C/T	8	38445203		
rs642961	IRF6	C/T	1	208055893	Interferon regulatory factor 6	1: key role in keratinocyte development; 2: consistent evidences of association to NSCL/P throughout many studies [17], [23], [44], [45].
rs2236907	IRF6	G/T	1	208038251		

All the positions are using NCBI build 36.3 as reference. Chr chromosome. The two alleles in a SNP are given in the format of (wild type/derived type).

**Figure 1:**
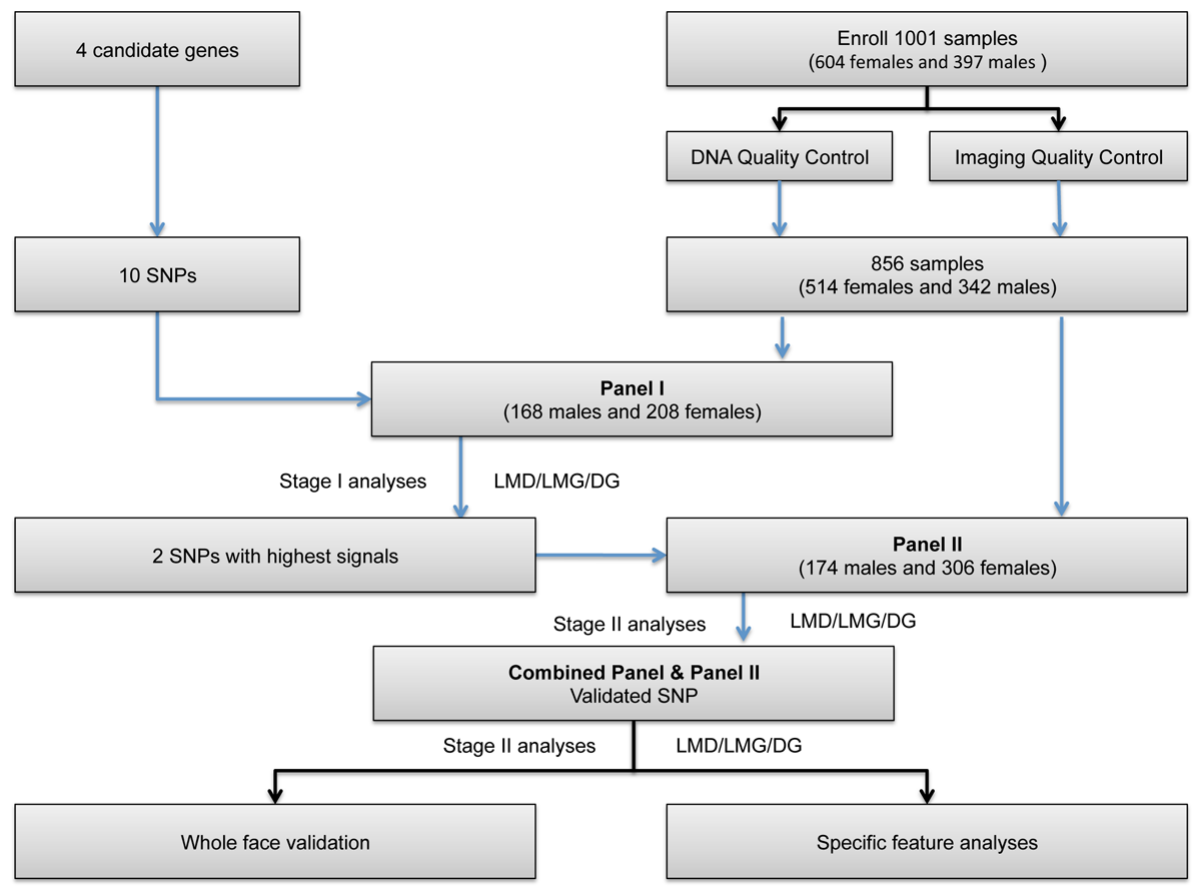
The flow chart of the whole analysis.

**Figure 2:**
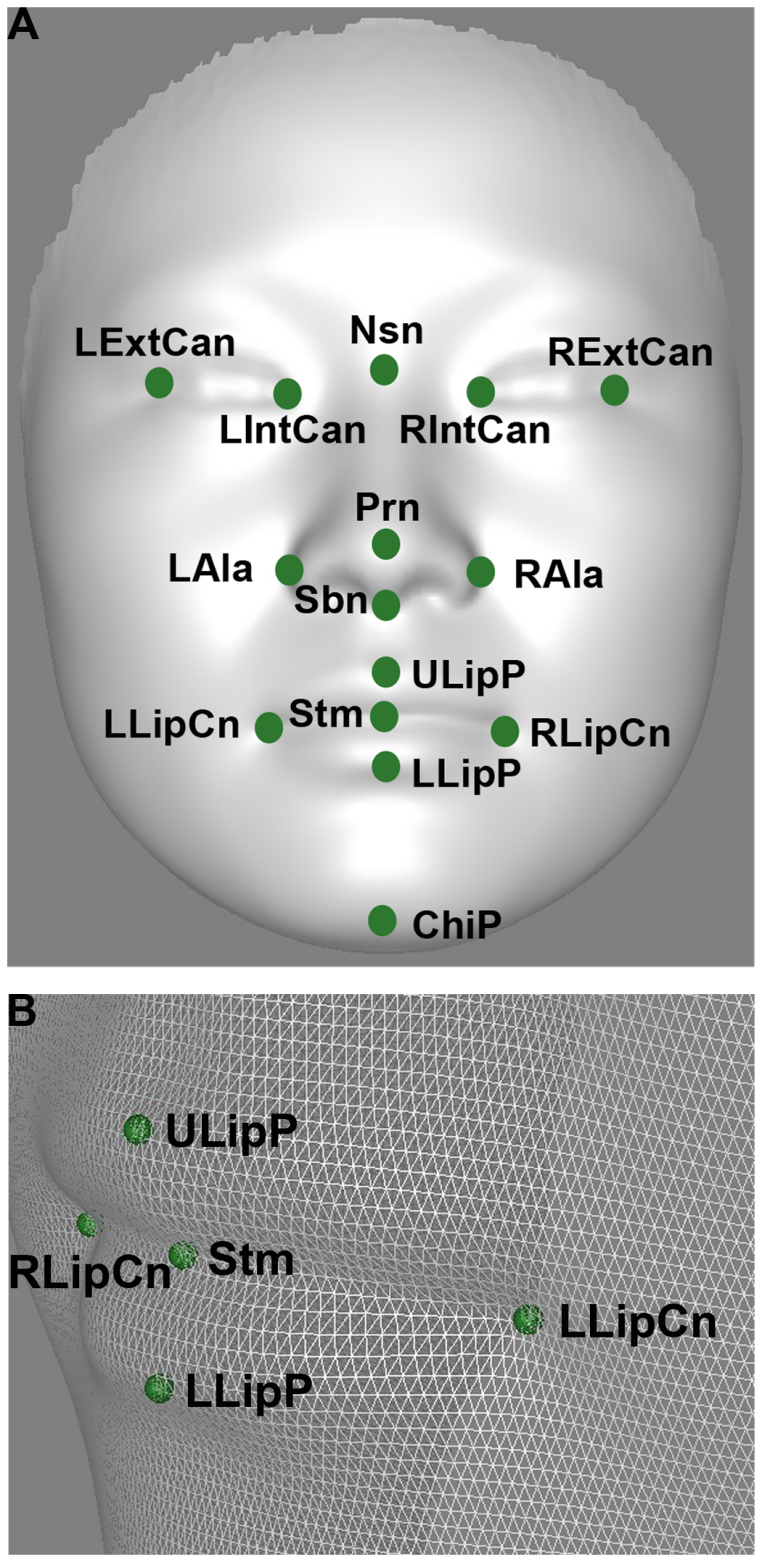
The 15 facial landmarks extracted from 3D imaging. A, An average face from the population is used for illustration. B, mouth part of average face showing the mesh of the 3D facial imaging. The abbreviation for landmarks: Left external canthus (LExtCan); Left internal canthus (LIntCan); Right internal canthus (RIntCan); Right external canthus (RExtCan); Pronasale (Prn); Nasion point (Nsn); Left Alare (LAla); Right Alare (RAla); Subnasale (Sbn); Right lip corner (RLipCn); Left lip corner (LLipCn); Stomion (Stm); Upper lip point (ULipP); Lower lip point (LLipP); Chin point (ChiP).

The genetic effects of these SNPs were examined in a Han Chinese population from Taizhou, Jiangsu province on the east coast of China. The complete work flow is summarized in figure 1. In total 1001 self-reported Han Chinese individuals were enrolled in the analyses (604 females and 397 males), with an age range of 17∼25 years. Saliva was collected to obtain DNA. For the phenotype data, we collected high resolution 3D facial images for each individual. Facial images were automatically annotated with 15 salient landmarks (see Fig. 2 for the full list of the landmarks), using a novel landmark recognition method (see Methods) [39]. This was followed by HDFR that resulted in 32,251 mathematically derived marker points, which were corresponded one to one across all individuals (see Methods) [39]. The facial shape phenotypes were represented with three different schemes. In the first scheme, the Euclidean distances between pairs of the landmarks were taken as phenotypes, and hereafter collectively referred to as the landmark-distance (LMD) data. In the second scheme, the 15 landmarks of different individuals were first superimposed into a common coordinate system, by partial general procrustes analysis (PGPA) (see methods) [37]. PGPA removes the differences in location and rotation, while keeping the size and shape information. The coordinates of the aligned landmarks were thus used as the second type of phenotypes, hereafter referred to as landmark-geometric data (LMG). In the third scheme, all the 32,251 marker points were used to describe the phenotypes. The marker points were similarly superimposed onto a common 3D space using PGPA, and the coordinate vectors specified a phenotype data space of 32,251×3 = 96,753 dimensions. This data is hereafter referred to as dense-geometric (DG) data.

As sampling was carried out during winter time, many 3D images were affected by the participants' collared clothing, especially around the upper neck and lower jaw area. Furthermore, heavy facial hair in males caused defects and holes in some surface meshes. During quality control, the images with obvious caveats were removed from further analysis (105 individuals). 40 individuals were further removed due to the poor DNA quality (uv light absorption A_260/280_<1.6 or total DNA quantity lower than 300 ng). In the end, 856 individuals were successfully processed for their 3D images and have corresponding DNA. We carried out the genetic association study in two stages. The individuals of the original cohort were randomly assigned to 2 panels: panel I included 376 individuals (168 males and 208 females), and panel II included 480 individuals (174 males, 306 females). Tests were carried out separately for different genders. In stage I, all 10 candidate SNPs were genotyped for panel I. Then in stage II, the markers that showed preliminary evidence of correlation were validated using panel II. For stage I analysis, individuals were assigned into 3 possible genotype groups for each SNP. None of these SNPs deviated significantly from the Hardy-Weinberg equilibrium. For the LMD data, the landmark-distances were tested for association with SNP genotypes using the Tukey's honestly significant difference test (Tukey's HSD test). Of the total 105 possible pairwise distances, 6 departure from normal distribution according to Shapiro-Wilk normality test. As normality is required in Tukey's HSD test, these phenotypes were removed from further analysis. For the remaining 99 phenotypes, the raw p values were calculated and corrected for multiple-testing with 10,000 permutations (see Methods). Table 2 shows the summary of the preliminary association signals. Several SNPs demonstrated some preliminary association signals in terms of nominal Tukey test p value (p value<0.01) (Table 2). In particular SNPs rs642961and rs6184, showed enriched signals (Table 2). For SNP rs642961, many signals appeared in females between the mutant (*TT*) and the other two groups *CC* and *CT*. Interestingly, the strongest signals seemed to all point to the area around mouth and lower nose area. The distances between the right/left lip corners and the right/left alare (RLipCn – RAla and LLipCn – LAla) had nominal Tukey test p values between 0.002∼0.004 in both the *CC/TT* and *CT/TT* comparisons (Table 2). The distance between the upper lip point and lower lip point (ULipP-LLipP) also suggested potential shape difference between the *CC* and *TT* groups (nominal p value = 0.0023, Table 2). The suggestive involvement of this SNP with mouth shape is consistent with the known role the host gene *IRF6* plays in NSCL/P [17], [23], [44], [45]. SNP rs6180 and rs6184 both showed some signals in males, which seemed to mainly involve the two lip corners and their relative positions to the middle line landmarks such as Pronasale, Nasion, Subnasale, lower lip point and chin (Table 2). These phenotypes may suggest size differences in the lower face among different genotype groups, but the overall trend is not clear. However, after the permutation correction of the multiple testing, none of these phenotypes stood significant at the individual SNP level, before accounting for multiple SNPs and different genders (Table 2).

**Table 2:** Stage I tests based on Landmark distance (LMD) data.

SNP	Gene	Trait	AA:BB		AA:AB		BB:AB	
			Male	Female	Male	Female	Male	Female
rs642961C/T *	IRF6	LExtCan - RAla	1.00	0.0116	0.994	0.859	1.00	0.00818
			1	0.308	1	1	1	0.242
		RLipCn - RAla	0.844	0.00241	0.935	0.893	0.784	0.00195
			1	**0.096**	1	1	1	**0.0798**
		RLipCn - ULipP	0.604	0.00316	0.391	0.595	0.884	0.0185
			1	0.1187	1	1	1	0.417
		LLipCn - LAla	0.760	0.00269	0.952	0.992	0.829	0.00360
			1	0.104	1	1	1	0.129
		LAla - RAla	0.832	0.0487	0.985	0.173	0.869	0.00665
			1	0.708	1	0.979	1	0.209
		LAla - LLipP	0.977	0.00487	0.968	0.958	0.957	0.0109
			1	0.163	1	1	1	0.296
		ULipP - LLipP	0.478	0.00231	0.861	0.631	0.610	0.0133
			1	**0.0930**	1	1	1	0.337
rs2236907G/T		LLipCn – Sbn	0.588	0.256	0.879	0.00839	0.158	0.226
			1	0.998	1	0.259	0.975	0.996
rs6180A/C	GHR	RLipCn - Prn	0.369	0.732	0.371	0.811	0.00352	0.204
			1	1	1	1	0.126	0.992
		RLipCn - LLipP	0.327	0.981	0.413	0.922	0.00328	0.971
			1	1	1	1	0.118	1
		RLipCn - Sbn	0.0806	0.389	0.923	1.00	0.00376	0.206
			0.872	1.00	1	1	0.134	0.993
		RLipCn - ChiP	0.621	0.988	0.148	0.894	0.00299	0.739
			1	1	0.971	1	0.110	1
		LAla - ChiP	0.623	0.963	0.172	0.970	0.00401	0.999
			1	1	0.983	1	0.141	1
rs6184C/A		RLipCn - Prn	0.00887	0.687	0.00152	0.611	0.184	0.895
			0.255	1	**0.0662**	1	0.984	1
		RLipCn - Nsn	0.0145	0.341	0.00969	0.243	0.805	0.715
			0.354	1.00	0.272	0.996	1	1
		RLipCn - Stm	0.0223	0.587	0.00377	0.783	0.150	0.673
			0.468	1	0.135	1	0.966	1
		RLipCn - ULipP	0.0184	0.415	0.00338	0.745	0.179	0.305
			0.415	1	0.122	1	0.982	0.999
		RLipCn - LLipP	0.0249	0.735	0.00453	0.856	0.166	0.305
			0.494	1	0.157	1	0.976	1
		LLipCn - Prn	0.0257	0.881	0.00919	0.882	0.441	0.999
			0.503	1	0.260	1	0.966	1
		LLipCn - Nsn	0.0153	0.311	0.00831	0.256	0.692	0.865
			0.368	0.999	0.243	0.997	1	1
		Nsn - Stm	0.0179	0.292	0.00970	0.241	0.689	0.867
			0.406	0.999	0.272	0.996	1	1
rs6569759G/A	ENPP1	RIntCan- ULipP	0.749	0.00400	0.394	0.00493	0.357	0.998
			1	0.139	1.00	0.163	1.00	1
		RExtCan - ULipP	0.910	0.0160	0.547	0.00616	0.301	0.736
			1	0.382	1	0.192	0.999	1

*p* values lower than 0.1 were marked in bold. The abbreviation for landmarks: Left external canthus (LExtCan); Left internal canthus (LIntCan); Right internal canthus (RIntCan); Right external canthus (RExtCan); Pronasale (Prn); Nasion point (Nsn); Left Alare (LAla); Right Alare (RAla); Subnasale (Sbn); Right lip corner (RLipCn); Left lip corner (LLipCn); Lip center (Stm); Upper lip point (ULipP); Lower lip point (LLipP); Chin point (ChiP). For each LMD comparison, the first row is the raw Tukey p value, and the second row is the p value corrected by permutation. All the comparisons with raw p value below 0.01 were listed. The permutation

For the LMG and DG data, we did the test for the whole geometric shapes, in a similar way to that previously described [37]. Briefly, the mean shapes were computed for each genotype group (see Methods), and the mutual distances between any two mean shapes were calculated. The mutual distance was calculated as the point-wise Procrustes distances (PPD), which is the Procrustes distance normalized by the number of landmarks/marker-points (see Methods). PPD distance can be directly compared between the LMG and DG data. The observed PPD distances were compared to 5000 random permutations to calculate empirical *p* values (see Methods). This procedure resulted in a single empirical *p* value for each comparison. The geometric permutation tests indicated that several SNPs had a nominal significance of association in stage I, and these signals were highly consistent between the LMG and DG data (Table 3, Table S1). To facilitate the visualization of the detailed differences, we also calculated the point-wise Euclidean distances between the mean shapes of the DG data, plotted as color gradients across the whole face (see Methods, Fig. S1). In gene *IRF6*, two SNPs rs2236907 and rs642961 exhibited moderate evidence of association. rs2236907 showed preliminary signals in both males and females (Table 3). However, a stronger association was found for rs642961 in females, where the *CC* and *CT* types both differ substantially from the *TT* type. The LMG tests had lower *p* values (nominal *p* = 0.005 and 0.006 for the *CC/TT* and *CT/TT* comparisons) than the DG tests (nominal *p* = 0.04 and 0.02 for the *CC/TT* and *CT/TT* comparisons) in both comparisons. Color gradient plots reveal that the major changes occur around the lips (Fig. S1A). The *GHR* SNP rs6184 showed some potential association between *CC* and *AA* in males (Table 3, Fig. S1 J). Two SNPs in the *ENPP1* gene, rs6925433 and rs7773292 that were previously found to be associated with vertical upper face measurements in the Chuvashian population [33], also showed potential association signals in our data (Table 3). The preliminary signals were in males, although rs7773292 may be involved in forehead shape (Fig. S1B), whereas SNP rs6925433 may be related to the chin area (Fig. S1D). SNP rs7773292 had the second strongest association signal among all the 10 markers, with the corresponding nominal *p* values scoring 0.015 and 0.034 in LMG and DG data respectively (Table 3). The highly consistent pattern of *p* values between LMG and DG suggests that the 15 landmarks for the LMG data captured the total facial shape variation well. It is also worth noting that signals based on LMD data (rs642961, rs2236907 and rs6184) overlapped substantially with those from LMG and DG data, suggesting a general compatibility among the three different schemes. The signals from geometric tests (LMG, DG) were stronger than those of LMD, as their *p* values stood nominally significant at individual SNP level, whereas none of the LMD tests passed the single SNP significance level after permutation correction. Globally, none of the LMG/DG proved significant after Bonfferoni correction assuming 60 independent tests (3 genotypes and 2 genders ×10 SNPs).

**Table 3:** The 5 SNPs of marginal significance in the first stage tests.

SNP	Female						Male					
	AA:BB		AA:AB		BB:AB		AA:BB		AA:AB		BB:AB	
	PPD	P	PPD	P	PPD	P	PPD	P	PPD	P	PPD	P
rs642961C/T*	**1.16**	**0.0404**	0.064	0.845	**1.17**	**0.0208**	1.93	0.222	2.09	0.165	0.0825	0.806
	**1.14**	**0.00536**	0.0496	0.922	**1.07**	**0.00624**	1.19	0.365	0.0476	0.972	1.17	0.347
rs2236907G/T	0.144	0.665	**0.317**	**0.0602**	0.139	0.327	0.491	0.147	**0.526**	**0.058**	0.080	0.839
	2.73	0.57	**3.61**	**0.0566**	2.34	0.394	3.65	0.371	3.34	0.323	2.25	0.657
rs6184C/A	1.00	0.522	0.166	0.484	1.43	0.307	**2.62**	**0.084**	0.118	0.653	2.25	0.212
	0.606	0.809	0.177	0.309	0.869	0.642	**2.15**	**0.026**	0.086	0.784	2.08	0.103
rs6925433A/G	0.0853	0.900	0.0634	0.925	0.117	0.481	**0.543**	**0.065**	**0.491**	**0.060**	0.113	0.680
	0.0578	0.975	0.0476	0.971	0.0826	0.661	**0.435**	**0.034**	0.296	0.118	0.174	0.261
rs7773292C/T	0.193	0.402	0.0840	0.817	0.0861	0.742	**0.482**	**0.075**	**0.566**	**0.034**	0.322	0.114
	0.121	0.681	0.0736	0.827	0.0680	0.842	**0.339**	**0.075**	**0.421**	**0.015**	0.236	0.101

“A”, the mutant is denoted by “B”. Tests that passed the nominal significance level of 0.1 are marked in bold. The significance level after Bonferroni correction is 0.00083. The two alleles in a SNP are given in the format of (wild type/derived type),e.g. (C/T), where the wild type is denoted by

**Figure 3:**
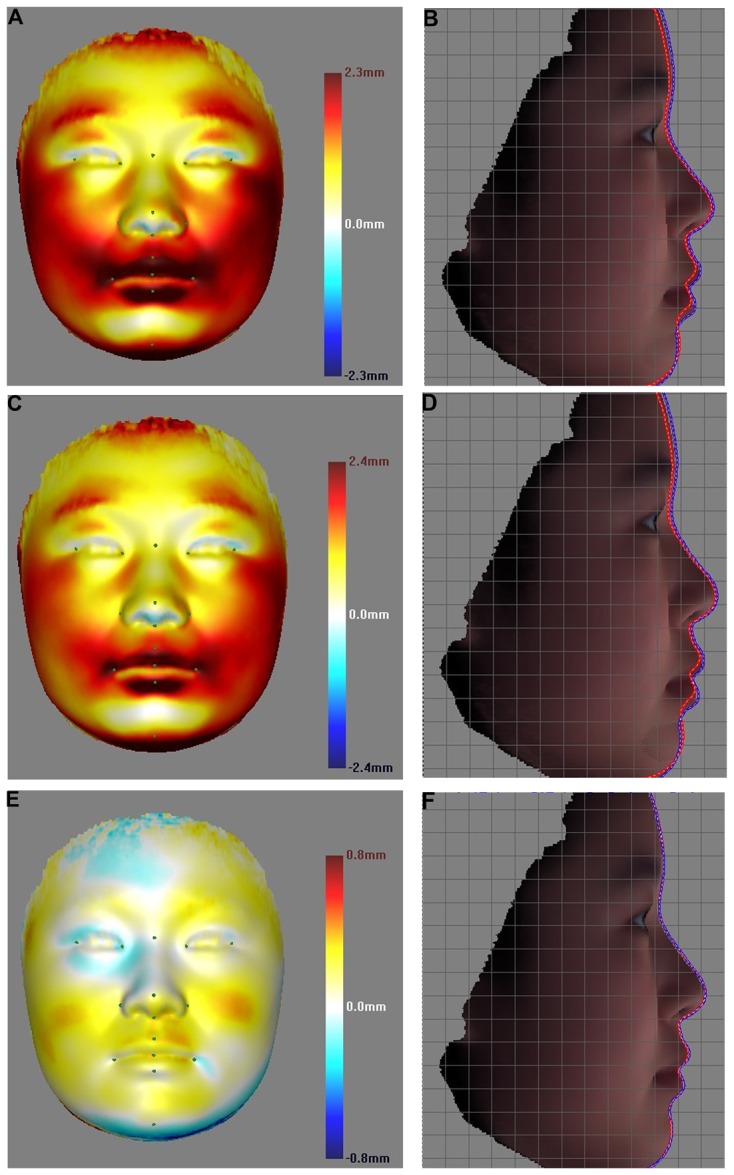
Facial shape comparisons among the genotype female groups of rs642961. The average shapes of the different genotype groups of rs642961 were compared pair-wisely, either for the point-wise distances, represented as color gradients in the left column; or for the contrast of the facial profile lines in the right column. The first, second and third rows denote the comparisons of CC/TT, CT/TT and CC/CT, respectively. In A, C and E, the higher intensity of the color gradient indicates greater point-wise distance. The first genotype group average face as the reference face (e.g, CC in the CC:TT comparison). The white color indicate no difference between reference face and compared face. The cold (or warm) colors indicate that the average shape of the reference face in a comparison is inside (or outside) of the compared face. In B, D and F, the red profile line is the average shape of the first genotype, and the blue line denotes the second genotype.

Since the geometric tests gave obviously stronger association signals than the LMD tests, we chose the candidate SNPs based on the LMG/DG results for further re-validation. The two SNPs rs642961 and rs7773292, from genes *IRF6* and *ENPP1* respectively exhibited the most prominent signals in stage I tests, and were selected to be revalidated in sample panel II. The same tests as in stage I were carried out either solely with panel II or with the combined panel of I and II together. The LMD data showed strong associations between rs642961 and several distances involving mouth landmarks, e.g. LLipP, ULipP and Stm (Table S2). In particular in panel II, six pairwise distances, RAla-Stm, RAla- LLipP, LAla-LLipP, Stm-Sbn, ULipP-Sbn and LLipP-Sbn remained significant or marginally significant for the *CC/TT* and *TT/CT* comparisons (corrected significance level 0.01, Table S2). For the combined panel, the distance between LAla and LLipP gave corrected *p* values of 2.0×10^−4^ and 3.0×10^−4^ respectively for the *CC/TT* and *CT/TT* comparisons (Table S2). Association signals in rs642961 were much more significant when the tests were carried out using the geometric data (Table 4). In panel II alone, the females remained significant in the *CC/TT* comparison (corrected *p* values 0.022 and 0.011), and marginally significant in the *CT/TT* comparison (corrected *p* values 0.089 and 0.054) after correcting for 12 tests (2 SNPs×6 comparisons). The same 4 comparisons were more significant in the combined panel (corrected *p* values 0.001∼0.065) after correcting for all 60 possible tests with 10 SNPs (Table 4). The color gradient plots based on the dense geometric data in combined panel revealed substantial facial morphological differences between rs642961 *TT* and the other two genotypes (Fig. 3 A,C,E), which were also highly consistent with the patterns revealed in panel I (Fig. S1 A). These plots clearly show that the strongest changes occur around the mouth region. The comparison of the face profile lines revealed that the *TT* carriers on average had a slightly elevated forehead, as well as thicker and more protrusive (2–3 mm outwards) lips, than the other two genotypes (Fig. 3 B, D, F). However, the signals from rs7773292 completely disappeared in all stage II tests (Table S3), suggesting a possible false positive signal.

**Table 4:** Validation of the association signals in rs642961.

rs642961(C/T)		CC:TT		CC:CT		TT:CT	
	Data type	PPD	P value	PPD	P value	PPD	P value
Female							
Panel I+II	DG	**1.61**	**0.0004**	0.0336	0.71	1.57	0.0011
	LMG	**1.38**	**2e-05**	0.0315	0.63	**1.28**	**0.00018**
^m^Panel I+II	DG	**1.90**	**<1e-05**	0.030	0.45	**1.74**	**6e-05**
	LMG	**1.48**	**<1e-05**	0.0251	0.44	**1.45**	**0.0007**
Panel II	DG	**2.58**	**0.0019**	0.070	0.48	2.65	0.0074
	LMG	**1.90**	**0.00098**	0.0566	0.49	1.97	0.0045
^m^Panel II	DG	**2.39**	**0.00050**	0.0811	0.17	**2.69**	**0.00046**
	LMG	**2.30**	**7e-05**	0.0560	0.22	**2.69**	**0.00038**
Male							
Panel I+II	DG	0.357	0.80	0.0563	0.65	0.397	0.77
	LMG	0.300	0.86	0.0349	0.89	0.321	0.85
^m^Panel I+II	DG	0.281	0.656	0.0295	0.75	0.326	0.59
	LMG	0.372	0.326	0.0204	0.82	0.353	0.41
Panel II	DG	0.888	0.62	0.252	0.10	1.52	0.36
	LMG	0.786	0.65	0.157	0.19	1.02	0.51
^m^Panel II	DG	1.18	0.210	0.126	0.27	1.33	0.23
	LMG	1.29	0.069	0.0641	0.51	1.26	0.11

+II). The tests for mouth region only are marked as ^m^.The significance level after Bonferroni correction is 0.0042 for panel II and 0.00083 for the combined panel (I+II). The p values that remain significant after correction are marked in bold. The results were based on 100,000 permutations. Permutation tests were performed in either panel II or in the combined panel (Panel I

To investigate the mouth shape changes associated with SNP rs642961 in more details, we extracted the mouth DG data from the whole face by retrieving a defined set of marker points for the mouth. The 5 mouth landmarks (LLipCn, RLipCn, ULipP, Stm, LLipP) were also extracted to compose the mouth LMG data. The landmark-distance analyses were not repeated as they remained the same despite the extraction of the mouth data. Geometric permutation tests were conducted as before for the mouth LMG and DG data. In general, the results seemed to be much more significant than the corresponding whole face comparisons (Table 4). In panel II, the extreme nominal *p* value of 7×10^−5^ (corrected *p* = 0.00084) occurred between *CC* and *TT* in females in the LMG data. In the combined panel, the *CC/TT* comparison in females had the minimum *p* value of 1×10^−5^ (corrected *p* = 0.00012) in both the LMG and DG data. It should be noted that these p values for mouth region do not indicate any formal statistical significance as they were conditional on the prior information of the genetic association in mouth shape. Nonetheless, the extreme *p* values suggested there are substantial impacts of genetic variants on normal mouth shape variation. One potential problem that may affect the mouth shape analysis is the stomion point. Stomion is the central point between the upper and lower lips. None-neutral expressions or open mouth may induce altered distances between stomion and other mouth landmarks, therefore confound the association signals. Our image dataset has been carefully screened for such cases. In order to formally test the impacts, we removed stomion from the landmark set, and re-ran the image registration procedure and the LMG/DG analyses for SNP rs642961. As can be seen in Table S4, the results remained largely unchanged, indicating that our results were not confounded by stomion variation. Another potential confounding factor is age, as facial appearance changes during the time course of aging. We carried out formal tests to examine whether there were non-negligible age effects in our sample. As age 18 and 21 seemed to define the tails of the sample age distributions (Fig. S2), we grouped the individuals of 18 years or younger, and of 21 years or older, from the combined panel. The average shape difference was tested on the DG data using permutation (see methods). Neither test was significant (p value = 0.267 for female; and 0.576 for male). The same test was performed between other age groups, and also did not reveal any significant age/face interactions. This suggests that age has little impact to the overall analyses in this study.

**Figure 4:**
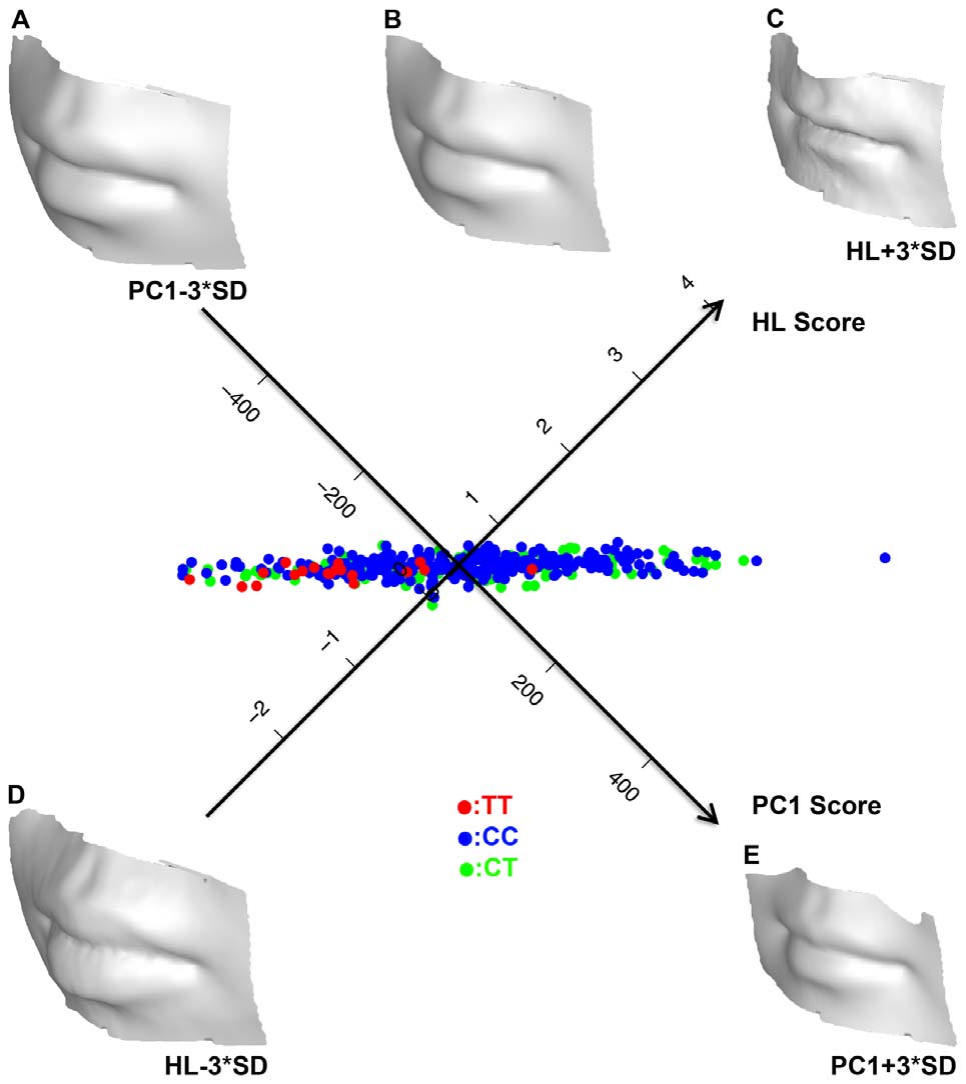
The distribution of individual mouth shapes along the PC1 mode and mean hyperline in females. Individual mouth shape was projected onto the two-dimensional space defined by the PC1 mode and the mean hyperline. Each point is annotated for the corresponding genotype. The PC1 and hyperline axes were plotted to intercept at the centroid (76.58, 0.45) of all female data points. The average mouth shape of all females (B) is plus or minus 3 times the sample standard deviation on either the PC1 mode or the hyperline. PC1+ (E) and PC1− (A) are the average shape +/− 3SD in PC1, and HL+ (C) and HL− (D) are the average shape +/− 3SD on the hyperline.

The mouth shape changes among different genotypes seem to involve complex shape changes, thus we performed further high-dimensional data analyses to describe such changes. In the following analyses, we used the combined female panel unless otherwise specified. We first carried out principle component analysis (PCA, see Methods) on both the LMG and DG data. In the DG data, the first PC mode best distinguished the *TT* and *CC* genotypes (t-test nominal *p* = 1.3×10^−6^), and the *TT/CT* comparison was also highly significant (t-test nominal *p* = 1.8×10^−6^) on this PC. The first PC from the LMG data revealed similarly strong differences in the *TT/CC* (t-test nominal *p* = 2.7×10^−6^) and *TT/CT* (t-test nominal *p* = 2.2×10^−6^) comparison. The large differences between *TT* and the other two genotypes and the little difference between *CC* and *CT* suggested that this locus may follow a dominant model. To formally test this, we constructed an additive model and a dominant model based on the standard linear model (see Methods). The additive model did not suggest any statistical significance, whereas the dominant model was highly significant both with the LMG (nominal *p* = 1×10^−6^) and the DG data (nominal *p = *6.8×10^−6^). Based on the dominant model, the genotypes of rs642961 explained a substantial proportion of the total variance (5.24% in the LMG data; 4.46% in the DG data) in PC1. Interestingly, when we tested these two models in a combined panel that included both males and females, the additive model remained insignificant, and the dominant model also became only marginally significant (nominal *p* = 0.003 in the LMG data; nominal *p* = 0.0159 in the DG data). This suggests that the effect of *TT* is female specific. To extract the facial pattern that best distinguishes *TT* from the other genotypes, we further carried out a simple linear discriminant analysis (LDA). As a hyperline that transects the mean points of *TT* and *CC* groups would best separate these two groups, this line was defined as a new data axis onto which individual data points were projected to generate hyperline (HL) scores. The HL scores were plotted against the PC1 scores to visualize data distribution (Fig. 4). As can be seen from Fig. 4, the distributions on PC and HL are highly correlated (r^2^ = 0.97). The *TT* distribution differed substantially from that of *CC* and *CT*. Specifically, the average PC1 score of 0 found 18 of the 19 *TT* individuals at the minus side; similarly, the average HL score of 0.444 had 18 out of 19 *TT* individuals at the minus side. To visualize the mouth shape changes, we transformed the mean shape (Fig. 4B) by adding or subtracting 3 standard deviations along either dimension as: ***s_t_*** = ***s_m_***±3*σ_v_* ***v***, where ***s_t_*** was the transformed shape, ***s_m_*** the average shape, ***v*** the Eigen vector of the dimension and *σ_v_* the standard deviation. The resulting shapes were defined as PC1+, PC1−, HL+ and HL− respectively in Fig. 4. The PC1− shape (Fig. 4A), which represents the trend for *TT*, has more protrusive and thicker lips compared to the finer and thinner lips in the PC1+ shape (Fig. 4E). The whole mouth region of PC1− is also more prominent and bigger than that of PC1+. Consistent with the high correlation between HL and PC1, the face models along the HL dimension reveal similar shape changes. (Fig. 4).

## Discussion

To the authors' knowledge, this study is the first to use high resolution face image registration to test the genetic association for common facial variation. Human face is a highly complex geometric surface. The simple inter-landmark distances used in previous studies may have over-simplified the common variation of human faces. As the high throughput acquisition of high content 3D image data becomes easier, methods based on shape geometric information, especially of high definition, become increasingly necessary to enable comprehensive and fully quantitative analyses of the complex facial traits. Based on high density 3D face registration, we compared three different schemes of phenotype during tests of genetic association, including LMD, LMG and the high resolution geometric data DG. We found that, in general, the three schemes produced consistent signals for the candidate SNPs. In the stage I test, the LMD method had only moderate association signals, mainly due to the large number of tests. The 15 landmarks gave rise to 105 possible tests in each genotype comparison (Table 2). One strategy to reduce the number of tests is to use only the essential distances, e.g. the conventional craniometrical measures that correspond to obvious anatomical structures. However, this risks missing the strongest signals. The other major problem with distance data is the difficulty in perceiving the underlying shape changes. For example in stage I, SNP rs642961 did not show a clear involvement with mouth shape changes in the LMD tests (Table 2). However, such an involvement was already quite clear on the DG comparison in stage I (Fig. S1). The LMD method seemed to improve both in the test power as well as the inference of shape changes (most significant landmark-distances involved the mouth landmarks) when larger sample sizes were used in stage II tests.

The two geometric schemes were generally found to give stronger association signals, implying better statistical power for the geometric methods. This may be due partially to the fact that the geometric tests were carried out in one step, which avoided a complex test structure. Interestingly, the LMG data of only 15 landmarks showed highly consistent test signals with that based on DG data. This suggests that these 15 landmarks capture the majority of the normal facial morphological variation. When only shape difference is to be tested, the LMG method seems to provide better efficiency (given the smaller data involved) and potentially higher test power. However, the strong consistency between LMG and DG in the association signals attributed to rs642961 may be partially accounted for by the high landmark density around the mouth area (5 out of 15 chosen landmarks). Features with fewer landmarks would confer lower power in the LMG tests. On the other hand, the DG data has other unique advantages for shape change inference and modeling. We also show here that the point-wise distance distribution between the mean faces can highlight the areas of shape changes in high definition (Fig. 3), which can guide future in depth exploration. Furthermore, the effects of potential genetic factors may also be modeled visually as realistic 3D face images (Fig. 4). This may have hugely beneficial applications to forensic studies.

Variants in the *IRF6* gene have been found to predispose to the risk of NSCL/P [21]–[23], [47]. Nevertheless, a link between the *IRF6* gene and common facial variation has not been established. This is the first study that provides strong evidence that rs642961 also affects normal facial shape variation. In particular, *TT* individuals may have more protrusive and thicker lips (Fig. 4). Interestingly, such an effect is very likely female specific as the tests in males did not yield significant signals. Combination of both sexes in the dominant model test also suggested that males did not contribute to the association signals. This is not uncommon. For example, various types of NSCL/P have been found to have sex specific spectra, suggesting sex is an important epistatic factor in mouth morphogenesis [16], [48]. In females, the *TT* individuals showed a highly specific distribution on the plane defined by PC1 and hyperline (Fig. 4). This could be used during diagnosis to pre-screen the risk allele carriers by interpreting 3D pictures, therefore facilitating early prevention of NSCL/P.

We have also detected preliminary associations for other SNPs. Failure to validate these association signals does not exclude them from the candidate list of loci related to normal facial shape variation. Extended sample sizes as well as inclusion of samples from other populations will be needed to further increase our understanding of the genetics of human facial morphology.

## Materials and Methods

### Ethics statement

Sample collection in this study was carried out with the approval of the ethics committee of the Shanghai Institutes for Biological Science and in accordance with the standards of the Declaration of Helsinki. Written informed consent was obtained from every participant.

### Sample collection

In total 1001 combined individuals (604 females, 397 males) from self-reported Han Chinese were sampled from Taizhou, Jiangsu province. Age ranges were between 17 and 25 years. 2∼3 ml of saliva was collected from each participant for DNA extraction. Individuals with obvious health problems or any history of facial surgery were excluded from the study.

### DNA extraction and genotyping

Genomic DNA was extracted from saliva following a modified Phenol–chloroform protocol [49], then suspended in Tris-ethylenediaminetetraacetic acid (TE) buffer (0.01 m TrisHCl, 0.001 m EDTA, pH 8.0) and stored at −20°C. SNP genotyping was performed with the SNaPshot multiplex system on an ABI3130xl genetic analyzer by Genesky Biotech, Shanghai, China.

### High density 3D facial image collection and registration

The 3dMDface system (www.3dmd.com/3dMDface) was used to collect high-resolution 3D facial images from participants. This system captures 3D facial images at a speed of ∼1.5 milliseconds and a geometry accuracy of 0.2 mm RMS.

We applied a new approach to achieve high density point-wise registration across all 3D facial images [39]. In brief, 17 salient facial landmarks were first automatically annotated, based on the PCA projection of both texture and shape information. In this study, 15 out of the 17 landmarks were used in analysis (Fig. 2). Two earlobe points were excluded as many 3D images, mainly of female participants were missing parts of the ears due to the unbound long hair. Afterwards, a facial image of high quality and smooth shape surface was chosen as the reference, and its surface mesh was re-sampled for an even point distribution at a density of 1pixel per *mm* ^2^. The reference face was then warped to register every sample face by matching all the 15 landmarks, via a non-rigid thin-plate spline (TPS) transformation. The mesh points of the reference face were then projected to the sample surface to find their one-to-one correspondents. The projection points were then used to re-define the mesh of the sample facial surface [39]. As the same reference face was always used, the re-defined 3D point sets in the sample faces were also point-wisely corresponded across all samples. The non-rigid registration guided by the 15 landmarks also ensured that the point-wise correspondence was approximately anatomically homologous. Each sample face was represented by a set of 32,251 3D points, with their coordinate values stored in a 3×32,251 matrix. Generalized Procrustes analysis (GPA) was used to align the sample facial shapes into a common coordinate system. The details of the dense correspondence registration approach are described elsewhere [39].

### Pair-wise shape distance (PPD)

Assuming each shape is represented as a vector: ***s*** = [*x_1_, y_1_, z_1_, x_2_, y_2_, z_2_ … x_n_, y_n_, z_n_*], where *x_i_*, *y_i_*, *z_i_* stand for the x, y, z coordinates of the *i*th point. There are *n* points in total. For two shapes ***s*** and ***s′***, the squared Euclidean distance for the *i*th point is,
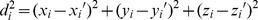
and the PPD is defined as:
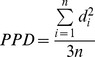



### Association tests

For the LMD data, in order to correct for the large number of sub-tests within each SNP, we performed a permutation procedure. For each of the 99 traits, raw p values were first calculated with Tukey's HSD test. A permutation procedure was used to correct the raw *p* values for multiple-testing. Briefly, the genotypes were reshuffled among the participants for 10,000 times and the Tukey's test was similarly carried out. The lowest *p* value from each permutation was combined to derive a null distribution. The empirical raw *p* values were then ranked against the null distribution to give the corrected permutation *p* values [50].

For the LMG and DG data, genotypes were randomly reshuffled among the individuals, and the PPD distances were calculated for the permutated genotype groups. 5000 permutations were carried out in stage I analyses and 100000 permutations in stage II analyses due to the much more significant P values. The PPD distribution under permutation was compared to the observed PPD value. The proportion of the permutation sets that had PPD values smaller than or equal to the observed PPD was taken as the nominal one-sided *p* value.

### PCA analysis

The prcomp function in the R statistics package was used for PCA analysis. An un-scaled PCA analysis was carried out, assuming equal variance for all points.

### Genetic model

We established both the dominant model and the additive model based on the standard linear model. The additive model was implemented by encoding genotypes as 0, 1 and 2. The dominant model was built by assuming *CC* and *CT* as 0 and *TT* as 1. Model test and analyses were conducted with the R statistics package.

## Supporting Information

Figure S1 **Mean facial shape comparisons for all 10 SNPs using Panel I.** The mean shapes of different genotype groups were compared pair-wisely. The point-wise distances are shown as color gradients. A higher intensity of color gradient indicates greater point-wise distance. The first genotype group average face as the reference face (e.g, *CC* in the *CC:TT* comparison). The white color indicates no difference between reference face and compared face. The cold(or warm) colors indicate that the average shape of the reference face in a comparison is inside (or outside) of the compared face. Genotypes with sample size less than 20 were marked in red, as their average face shapes and corresponding comparisons were less reliable.
(TIF)

Figure S2 **Age distribution in Taizhou population.**
(TIF)

Table S1 **The geometric permutation test of all the 10 SNPs in Panel I.**
(DOC)

Table S2 **Validation of the rs642961 signals based on LMD data.**
(DOC)

Table S3 **The results of the geometric permutation test of rs7773292 in the stage II analyses.**
(DOC)

Table S4 **Validation of the association signals in rs642961 after removing the stomion points.**
(DOC)
