# Supplementary figures and images for: Detecting Genetic Association of Common Human Facial Morphological Variation Using High Density 3D Image Registration
Source: PLoS Comput Biol. 2013 Dec 5;9(12):e1003375. doi: 10.1371/journal.pcbi.1003375 (PMC3854494; doi:10.1371/journal.pcbi.1003375)

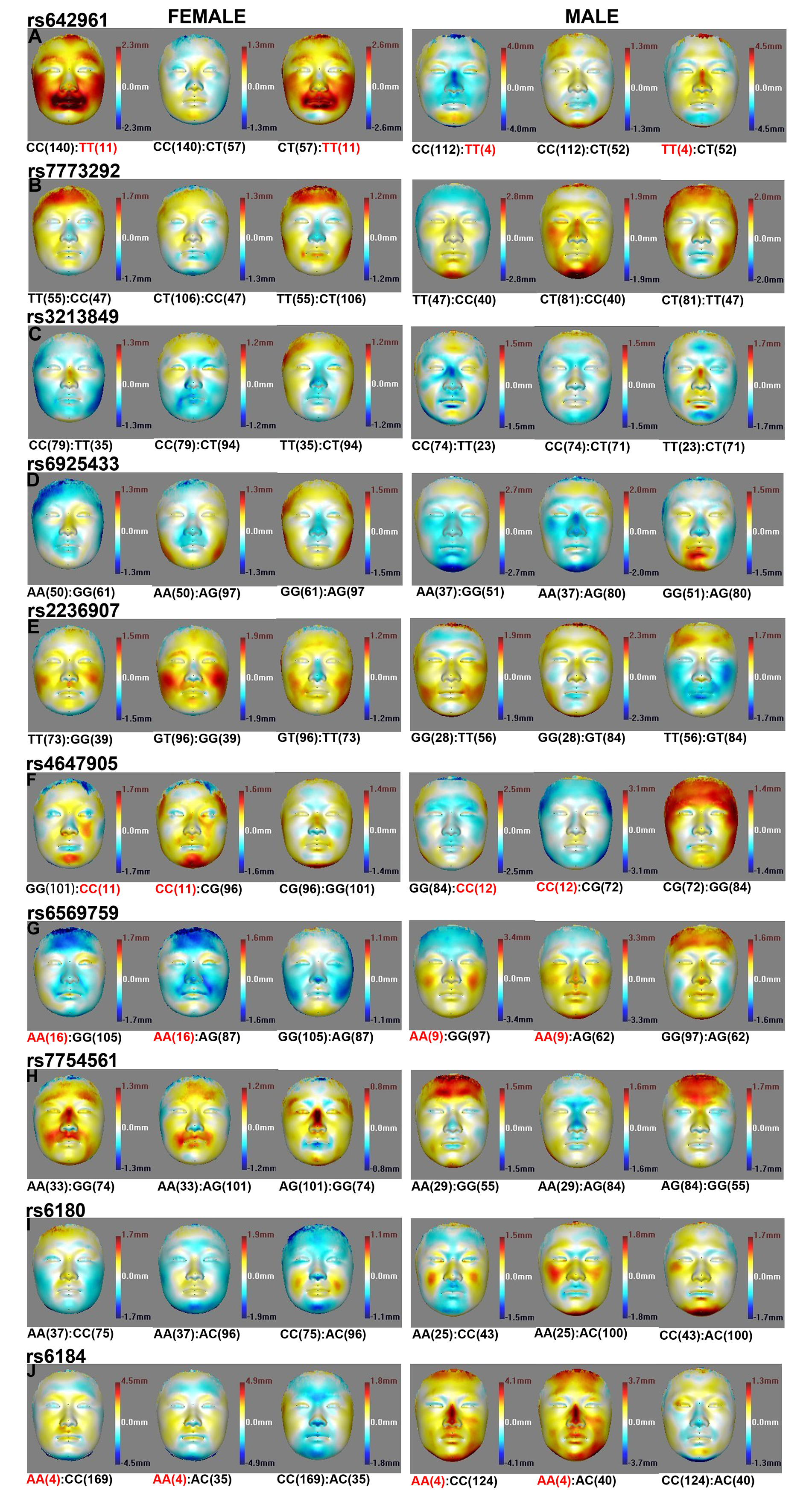

Supplement: Figure S1 — Mean facial shape comparisons for all 10 SNPs using Panel I. The mean shapes of different genotype groups were compared pair-wisely. The point-wise distances are shown as color gradients. A higher intensity of color gradient indicates greater point-wise distance. The first genotype group average face as the reference face (e.g, CC in the CC:TT comparison). The white color indicates no difference between reference face and compared face. The cold(or warm) colors indicate that the average shape of the reference face in a comparison is inside (or outside) of the compared face. Genotypes with sample size less than 20 were marked in red, as their average face shapes and corresponding comparisons were less reliable. (TIF) [file pcbi.1003375.s001.tif]

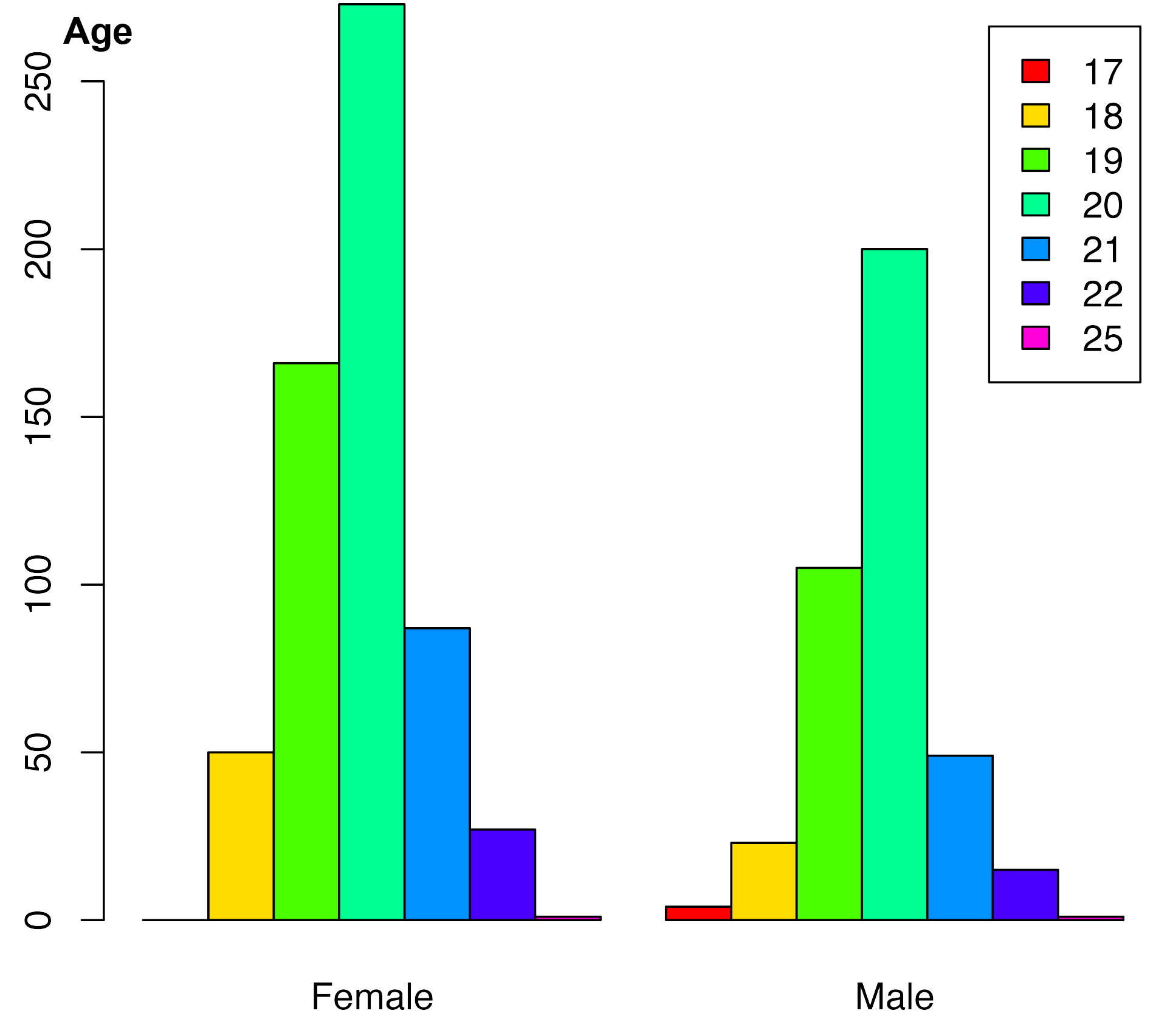

Supplement: Figure S2 — Age distribution in Taizhou population. (TIF) [file pcbi.1003375.s002.tif]
