# Supplementary material for: Detecting Genetic Association of Common Human Facial Morphological Variation Using High Density 3D Image Registration
Source: PLoS Comput Biol. 2013 Dec 5;9(12):e1003375. doi: 10.1371/journal.pcbi.1003375 (PMC3854494; doi:10.1371/journal.pcbi.1003375)
Supplement: Table S1 — The geometric permutation test of all the 10 SNPs in Panel I. (DOC) [file pcbi.1003375.s003.doc]

**Table S1. The geometric permutation test of all 10 SNPs in Panel I**

| SNP | Female | | | | | | Male | | | | | |
| --- | --- | --- | --- | --- | --- | --- | --- | --- | --- | --- | --- | --- |
|  | AA:BB |  | AA:AB |  | BB:AB |  | AA:BB |  | AA:AB |  | BB:AB |  |
|  | PPD | P | PPD | P | PPD | P | PPD | P | PPD | P | PPD | P |
| rs642961C/T * | 1.16 | 0.0404 | 0.064 | 0.845 | 1.17 | 0.0208 | 1.93 | 0.222 | 2.09 | 0.165 | 0.0825 | 0.806 |
|  | 1.14 | 0.00536 | 0.0496 | 0.922 | 1.07 | 0.00624 | 1.19 | 0.365 | 0.0476 | 0.972 | 1.17 | 0.347 |
| rs2236907G/T | 0.144 | 0.665 | 0.317 | 0.0602 | 0.139 | 0.327 | 0.491 | 0.147 | 0.526 | 0.058 | 0.080 | 0.839 |
|  | 2.73 | 0.57 | 3.61 | 0.0566 | 2.34 | 0.394 | 3.65 | 0.371 | 3.34 | 0.323 | 2.25 | 0.657 |
| rs6180A/C | 0.137 | 0.717 | 0.184 | 0.423 | 0.0691 | 0.777 | 0.230 | 0.651 | 0.444 | 0.123 | 0.230 | 0.276 |
|  | 0.104 | 0.816 | 0.123 | 0.631 | 0.0688 | 0.718 | 0.237 | 0.549 | 0.300 | 0.158 | 0.179 | 0.250 |
| rs6184C/A | 1.00 | 0.522 | 0.166 | 0.484 | 1.43 | 0.307 | 2.62 | 0.0846 | 0.118 | 0.653 | 2.25 | 0.212 |
|  | 0.606 | 0.809 | 0.177 | 0.309 | 0.869 | 0.642 | 2.15 | 0.0264 | 0.086 | 0.784 | 2.08 | 0.103 |
| rs3213849C/T | 0.248 | 0.282 | 0.207 | 0.0932 | 0.125 | 0.765 | 0.136 | 0.852 | 0.155 | 0.402 | 0.272 | 0.532 |
|  | 0.131 | 0.613 | 0.164 | 0.101 | 0.183 | 0.362 | 0.134 | 0.833 | 0.105 | 0.557 | 0.236 | 0.524 |
| rs4647905G/C | 0.227 | 0.928 | 0.123 | 0.295 | 0.340 | 0.623 | 0.305 | 0.718 | 0.238 | 0.133 | 0.569 | 0.361 |
|  | 0.241 | 0.845 | 0.113 | 0.195 | 0.273 | 0.763 | 0.296 | 0.638 | 0.135 | 0.289 | 0.311 | 0.635 |
| rs6569759G/A | 0.226 | 0.764 | 0.0513 | 0.883 | 0.261 | 0.655 | 0.652 | 0.412 | 0.167 | 0.317 | 0.991 | 0.260 |
|  | 0.232 | 0.645 | 0.0496 | 0.854 | 0.292 | 0.457 | 0.425 | 0.640 | 0.108 | 0.501 | 0.701 | 0.288 |
| rs6925433A/G | 0.0853 | 0.900 | 0.0634 | 0.925 | 0.117 | 0.481 | 0.543 | 0.065 | 0.491 | 0.0598 | 0.113 | 0.680 |
|  | 0.0578 | 0.975 | 0.0476 | 0.971 | 0.0826 | 0.661 | 0.435 | 0.034 | 0.296 | 0.118 | 0.174 | 0.261 |
| rs7754561G/A | 0.193 | 0.538 | 0.120 | 0.382 | 0.0630 | 0.987 | 0.175 | 0.641 | 0.132 | 0.559 | 0.106 | 0.892 |
|  | 0.196 | 0.423 | 0.111 | 0.316 | 0.091 | 0.864 | 0.0943 | 0.943 | 0.0797 | 0.832 | 0.0786 | 0.964 |
| rs7773292C/T | 0.193 | 0.402 | 0.0840 | 0.817 | 0.0861 | 0.742 | 0.482 | 0.0746 | 0.566 | 0.0344 | 0.322 | 0.114 |
|  | 0.121 | 0.681 | 0.0736 | 0.827 | 0.0680 | 0.842 | 0.339 | 0.0749 | 0.421 | 0.0154 | 0.236 | 0.101 |

* The two alleles in a SNP are given in the format of (wild type / derived type),e.g. (C/T), where the wild type is denoted by “A”, the mutant is denoted by “B”. For example, A/C means AA=AA, AB=AC, BB=CC. All tests were performed 5000 times. Each SNP has two rows, the first for DG test and the second for LMG test.
