# Supplementary material for: Detecting Genetic Association of Common Human Facial Morphological Variation Using High Density 3D Image Registration
Source: PLoS Comput Biol. 2013 Dec 5;9(12):e1003375. doi: 10.1371/journal.pcbi.1003375 (PMC3854494; doi:10.1371/journal.pcbi.1003375)
Supplement: Table S2 — Validation of the rs642961 signals based on LMD data. (DOC) [file pcbi.1003375.s004.doc]

**Table S2. Validation of the rs642961 signals based on LMD data.**

| SNP | Trait | AA:BB | | AA:AB | | BB:AB | |
| --- | --- | --- | --- | --- | --- | --- | --- |
|  |  | Male | Female | Male | Female | Male | Female |
| Panel II |  |  |  |  |  |  |  |
|  | LExtCan - RLipCn | 0.714 | 0.00465 | 0.0689 | 0.551 | 0.289 | 0.00173 |
|  |  | 1 | 0.138 | 0.810 | 1 | 0.999 | 0.0646 |
|  | LExtCan- LLipP | 0.378 | 0.00110 | 0.278 | 0.804 | 0.167 | 0.000664 |
|  |  | 1.00 | 0.0454 | 0.998 | 1 | 0.975 | 0.0289 |
|  | LIntCan - LLipP | 0.541 | 0.00140 | 0.526 | 0.631 | 0.345 | 0.000582 |
|  |  | 1 | 0.0548 | 1 | 1 | 1.00 | 0.0253 |
|  | RIntCan - Stm | 0.815 | 0.00431 | 0.547 | 0.728 | 0.612 | 0.00225 |
|  |  | 1 | 0.13 | 1 | 1 | 1 | 0.0779 |
|  | RIntCan - LLipP | 0.858 | 0.000752 | 0.551 | 0.759 | 0.664 | 0.000406 |
|  |  | 1 | 0.0326 | 1 | 1 | 1 | 0.0182 |
|  | RExtCan - LLipCn | 0.931 | 0.00541 | 0.205 | 0.567 | 0.627 | 0.00210 |
|  |  | 1 | 0.155 | 1.00 | 1 | 1 | 0.0735 |
|  | RExtCan - LLipP | 0.950 | 0.000981 | 0.619 | 0.798 | 0.814 | 0.000582 |
|  |  | 1 | 0.0406 | 1 | 1 | 1 | 0.0252 |
|  | Prn - Stm | 1.00 | 0.00144 | 0.659 | 0.858 | 0.950 | 0.000990 |
|  |  | 1 | 0.0557 | 1 | 1 | 1 | 0.041 |
|  | Prn - LLipP | 0.890 | 0.000251 | 0.706 | 0.877 | 0.755 | 0.000183 |
|  |  | 1 | 0.0111 | 1 | 1 | 1 | **0.0091** |
|  | Prn - ChiP | 0.668 | 0.00146 | 0.252 | 0.820 | 0.359 | 0.000915 |
|  |  | 01 | 0.0563 | 0.997 | 1 | 1.00 | 0.0385 |
|  | LAla - Stm | 0.397 | 0.000330 | 0.750 | 0.957 | 0.288 | 0.000674 |
|  |  | 1 | 0.0149 | 1 | 1 | 0.999 | 0.0297 |
|  | LAla - ULipP | 0.410 | 0.000462 | 0.937 | 0.944 | 0.362 | 0.000978 |
|  |  | 1 | 0.021 | 1 | 1 | 0.999 | 0.0405 |
|  | LAla - LLipP | 0.465 | 6.54e-05 | 0.629 | 0.972 | 0.312 | 7.09e-05 |
|  |  | 1 | **0.0042** | 1 | 1 | 0.999 | **0.0044** |
|  | RAla - Stm | 0.450 | 2.22e-05 | 0.679 | 0.972 | 0.312 | 4.91e-05 |
|  |  | 1 | **0.0016** | 1 | 1 | 1 | **0.0034** |
|  | RAla - ULipP | 0.856 | 0.000252 | 0.959 | 0.732 | 0.814 | 0.000967 |
|  |  | 1 | 0.0111 | 1 | 1 | 1 | 0.0404 |
|  | RAla - LLipP | 0.719 | 3.11e-05 | 0.713 | 0.998 | 0.565 | 5.31e-05 |
|  |  | 1 | **0.0023** | 1 | 1 | 1 | **0.0034** |
|  | Stm - Sbn | 0.665 | 1.62e-05 | 0.842 | 0.874 | 0.559 | 1.20e-05 |
|  |  | 1 | **0.0014** | 1 | 1 | 1 | **6.00e-04** |
|  | ULipP - Sbn | 0.874 | 3.70e-05 | 0.921 | 0.526 | 0.933 | 1.08e-05 |
|  |  | 1 | **0.0026** | 1 | 1 | 1 | **6.00e-04** |
|  | LLipP - Sbn | 0.595 | 1.91e-05 | 0.856 | 0.865 | 0.497 | 1.37e-05 |
|  |  | 1 | **0.0015** | 1 | 1 | 1 | **8.00e-04** |
|  | Sbn - ChiP | 0.438 | 0.000690 | 0.248 | 0.861 | 0.195 | 0.000477 |
|  |  | 1 | 0.0304 | 0.996 | 1 | 0.986 | 0.0215 |
| Panel I+II |  |  |  |  |  |  |  |
|  | LExtCan - LLipCn | 0.662 | 0.00140 | 0.354 | 0.833 | 0.384 | 0.00463 |
|  |  | 1 | 0.0481 | 1 | 1 | 0.9993 | 0.142 |
|  | LExtCan - Stm | 0.984 | 0.00123 | 0.571 | 0.996 | 0.863 | 0.00164 |
|  |  | 1 | 0.0424 | 1 | 1 | 1 | 0.0562 |
|  | LExtCan - LLipP | 0.971 | 5.56e-05 | 0.548 | 1.00 | 0.827 | 0.000154 |
|  |  | 1 | **0.0028** | 1 | 1 | 1 | **0.0054** |
|  | LIntCan - LLipP | 0.999 | 0.000308 | 0.734 | 0.896 | 0.950 | 0.000247 |
|  |  | 1 | 0.014 | 1 | 1 | 1 | 0.011 |
|  | RIntCan - LLipP | 0.954 | 0.000225 | 0.791 | 0.961 | 0.998 | 0.000245 |
|  |  | 1 | 0.01 | 1 | 1 | 1 | 0.011 |
|  | RExtCan - RLipCn | 0.969 | 0.000685 | 0.390 | 0.973 | 0.771 | 0.00148 |
|  |  | 1 | 0.0269 | 1 | 1 | 1 | 0.0505 |
|  | RExtCan - Stm | 0.943 | 0.000921 | 0.538 | 0.931 | 0.999 | 0.000819 |
|  |  | 1 | 0.0334 | 1 | 1 | 1 | 0.0305 |
|  | RExtCan - LLipP | 0.960 | 8.01e-05 | 0.659 | 0.928 | 0.999 | 7.51e-05 |
|  |  | 1 | **0.0039** | 1 | 1 | 1 | **0.0038** |
|  | RLipCn - RAla | 0.649 | 0.000185 | 0.924 | 0.306 | 0.581 | 0.00283 |
|  |  | 1 | **0.008** | 1 | 0.999 | 1 | 0.0925 |
|  | LLipCn - Prn | 0.275 | 0.000989 | 0.927 | 0.475 | 0.236 | 0.00741 |
|  |  | 0.998 | 0.0353 | 1 | 1 | 0.995 | 0.207 |
|  | Prn - LLipP | 0.841 | 0.000134 | 0.937 | 0.899 | 0.904 | 0.000110 |
|  |  | 1 | **0.0068** | 1 | 1 | 1 | **0.0056** |
|  | LAla- Stm | 0.686 | 5.46e-05 | 0.955 | 0.922 | 0.636 | 5.04e-05 |
|  |  | 1 | **0.0028** | 1 | 1 | 1 | **0.0027** |
|  | LAla - ULipP | 0.432 | 0.000152 | 0.997 | 0.999 | 0.466 | 0.000240 |
|  |  | 1 | **0.007** | 1 | 1 | 1 | 0.0107 |
|  | LAla - LLipP | 0.754 | 2.85e-06 | 0.895 | 0.982 | 0.670 | 4.31e-06 |
|  |  | 1 | **2.00e-04** | 1 | 1 | 1 | **3.00e-04** |
|  | RAla - Stm | 0.928 | 6.08e-05 | 0.996 | 0.977 | 0.921 | 0.000154 |
|  |  | 1 | **0.0032** | 1 | 1 | 1 | **0.0071** |
|  | RAla - ULipP | 0.790 | 0.000699 | 0.607 | 0.979 | 0.949 | 0.00145 |
|  |  | 1 | 0.027 | 1 | 1 | 1 | 0.0498 |
|  | RAla - LLipP | 0.958 | 8.00e-06 | 1.00 | 1 | 0.960 | 1.63e-05 |
|  |  | 1 | **6.00-04** | 1 | 1 | 1 | **0.0013** |
|  | Stm - Sbn | 0.848 | 0.000671 | 0.776 | 0.801 | 0.952 | 0.000392 |
|  |  | 1 | 0.0265 | 1 | 1 | 1 | 0.017 |
|  | ULipP - LLipP | 0.998 | 0.000201 | 0.621 | 0.837 | 0.961 | 0.000816 |
|  |  | 1 | **0.0091** | 1 | 1 | 1 | 0.0305 |
|  | LLipP- Sbn | 0.999 | 2.18e-06 | 0.895 | 0.904 | 0.992 | 1.91e-05 |
|  |  | 1 | **0.0015** | 1 | 1 | 1 | **0.0013** |

Top 20 traits of raw Tukey test p value in each panel were selected. For each LMD comparison, the first row is the raw Tukey p value, and the second row is the p value corrected by permutation. The permutation *p* values lower than 0.01 were marked in bold. The abrevation for landmarks: Left external canthus (LExtCan); Left internal canthus (LIntCan); Right internal canthus (RIntCan); Right external canthus (RExtCan); Pronasale (Prn); Nasion point (Nsn); Left Alare (LAla); Right Alare (RAla); Subnasale (Sbn); Right lip corner (RLipCn); Left lip corner (LLipCn); Stomion (Stm); Upper lip point (ULipP); Lower lip point (LLipP); Chin point (ChiP).
