# Supplementary material for: Detecting Genetic Association of Common Human Facial Morphological Variation Using High Density 3D Image Registration
Source: PLoS Comput Biol. 2013 Dec 5;9(12):e1003375. doi: 10.1371/journal.pcbi.1003375 (PMC3854494; doi:10.1371/journal.pcbi.1003375)
Supplement: Table S3 — The results of the geometric permutation test of rs7773292 in the stage II analyses. (DOC) [file pcbi.1003375.s005.doc]

**Table S3. The results of the geometric permutation test of rs7773292 in the stage II analyses**.

| rs7773292 | CC:TT | | CC:CT | | TT:CT | |
| --- | --- | --- | --- | --- | --- | --- |
|  | PPD | P value | PPD | P value | PPD | P value |
| Female |  |  |  |  |  |  |
| Panel I+II | 0.0829 | 0.424 | 0.0478 | 0.641 | 0.0295 | 0.917 |
| Panel II | 0.102 | 0.487 | 0.0795 | 0.469 | 0.0361 | 0.953 |
| Male |  |  |  |  |  |  |
| Panel I+II | 0.124 | 0.481 | 0.0763 | 0.607 | 0.0695 | 0.663 |
| Panel II | 0.0916 | 0.951 | 0.220 | 0.211 | 0.188 | 0.337 |
