# Supplementary material for: Detecting Genetic Association of Common Human Facial Morphological Variation Using High Density 3D Image Registration
Source: PLoS Comput Biol. 2013 Dec 5;9(12):e1003375. doi: 10.1371/journal.pcbi.1003375 (PMC3854494; doi:10.1371/journal.pcbi.1003375)
Supplement: Table S4 — Validation of the association signals in rs642961 after removing the stomion points. (DOC) [file pcbi.1003375.s006.doc]

**Table S4. Validation of the association signals in rs642961 after removing the stomion point.**

| rs642961(C/T) |  | CC:TT | | CC:CT | | TT:CT | |
| --- | --- | --- | --- | --- | --- | --- | --- |
|  | Data type | PPD | P value | PPD | P value | PPD | P value |
| Female |  |  |  |  |  |  |  |
| Panel I+II | DG | 1.93 | **9.00e-05** | 0.0306 | 0.778 | 1.81 | **6.00e-04** |
|  | LMG | 1.80 | **3.00e-05** | 0.0321 | 0.646 | 1.64 | **4.00e-05** |
| ^m^Panel I+II | DG | 2.23 | **1.00e-05** | 0.0292 | 0.485 | 2.04 | **1.00e-05** |
|  | LMG | 1.81 | **4.00e-05** | 0.0467 | 0.498 | 1.82 | **3.90e-04** |
| Panel II | DG | 3.25 | **4.70e-04** | 0.0647 | 0.569 | 3.22 | 0.00306 |
|  | LMG | 2.80 | **1.00e-04** | 0.0584 | 0.506 | 2.72 | 0.00189 |
| ^m^Panel II | DG | 3.13 | **4.00e-05** | 0.0620 | 0.303 | 3.32 | **1.50e-04** |
|  | LMG | 3.11 | **2.00e-05** | 0.0532 | 0.316 | 3.56 | 0.00179 |
| Male |  |  |  |  |  |  |  |
| Panel I+II | DG | 0.353 | 0.802 | 0.0621 | 0.570 | 0.382 | 0.785 |
|  | LMG | 0.277 | 0.906 | 0.0427 | 0.802 | 0.284 | 0.921 |
| ^m^Panel I+II | DG | 0.261 | 0.694 | 0.0311 | 0.721 | 0.293 | 0.664 |
|  | LMG | 0.345 | 0.457 | 0.0217 | 0.818 | 0.331 | 0.529 |
| Panel II | DG | 1.00 | 0.516 | 0.262 | 0.0856 | 1.59 | 0.336 |
|  | LMG | 0.870 | 0.602 | 0.0612 | 0.140 | 1.11 | 0.520 |
| ^m^Panel II | DG | 1.33 | 0.161 | 0.134 | 0.237 | 1.47 | 0.191 |
|  | LMG | 1.25 | 0.138 | 0.0773 | 0.483 | 1.31 | 0.118 |

All the permutation under 100000 random times. Permutation tests were performed in either panel II or in the combined panel (Panel I+II). The tests for mouth region only are marked as ^m^.The significance level after Bonferroni correction is 0.0042 for panel2 and 0.00083 for the combined panel (I+II). The p values that remain significant after correction are marked in bold.
